# Liver FoxO1 overexpression is positively associated with the degree of liver injury in cirrhotic patients

**DOI:** 10.1515/almed-2023-0014

**Published:** 2023-07-12

**Authors:** Esther Fernández-Galán, Silvia Sandalinas, Laura Macias-Muñoz, Irene Portolés, Jordi Ribera, Blai Morales-Romero, Montse Pauta, Gregori Casals, Loreto Boix, Wladimiro Jiménez, Manuel Morales-Ruiz

**Affiliations:** Biochemistry and Molecular Genetics Department-CDB, Hospital Clínic of Barcelona, Barcelona, Spain; Institut d’Investigacions Biomèdiques August Pi i Sunyer (IDIBAPS), Centro de Investigación Biomédica en Red de Enfermedades Hepáticas y Digestivas (CIBERehd), Barcelona, Spain; Commission for the Biochemical Assessment of Hepatic Disease-SEQCML, Barcelona, Spain; Barcelona Clinic Liver Cancer Group, Liver Unit, Hospital Clínic of Barcelona, Barcelona, Spain; Department of Biomedicine-Biochemistry Unit, Faculty of Medicine and Health Sciences, University of Barcelona, Barcelona, Spain

**Keywords:** Akt, chronic liver disease, FoxO1, liver cirrhosis, liver regeneration

## Abstract

**Objectives:**

Chronic liver disease and related complications, cirrhosis and hepatocellular carcinoma, are associated with high mortality. Curative treatments, partial hepatectomy or liver transplantation, have limited applicability in patients with cirrhosis due to the poor liver regenerative capacity. Thus, we need to find new diagnostic and therapeutic alternatives, to block the disease progression and to improve the survival of patients. In this context, preclinical studies have demonstrated the key role of the protein kinase B (Akt) in liver dysfunction, but the status of Akt and its targets in patients with chronic hepatopathy remains unknown. Aims: To determine the activation status of the Akt pathway and their association with liver functionality in cirrhotic patients.

**Methods:**

This retrospective study includes liver tissue samples from 36 hepatectomized patients with (n=27) and without (n=9) cirrhosis. Multiplex analysis of proteins involved in the Akt/mTOR pathway was performed using a Luminex panel and Western blot. Conventional liver function tests were determined in serum before resection surgery.

**Results:**

Akt and forkhead box protein O1 (FoxO1) are overexpressed in the liver of cirrhotic patients: (2.1 vs. 1.0 densitometric relative units (DRU); p<0.01, and 9.5 vs*.* 4.4 DRU; p<0.01, respectively). FoxO1 showed the best correlation with markers of liver injury (aspartate aminotransferase (ASAT): r=0.51, p<0.05; alanine aminotransferase (ALAT): r=0.49, p<0.05), and was the only enzyme in the Akt pathway identified as an independent predictor of ASAT and ALAT levels.

**Conclusions:**

The intrahepatic expression of FoxO1 could have clinical utility as a potential prognostic marker for patients with advanced liver disease.

## Introduction

Medical care related to liver disease is complex, and requires extensive outpatient consultations; the disease results in many hospitalizations and high mortality rates due to complications such as hepatocellular carcinoma (HCC) and cirrhosis. Moreover, these conditions fall in the diagnostic category that has experienced the second-highest growth in the last five years, as indicated by available data [1]. One of the most remarkable facts is that this illness affects an economically active population and therefore impacts the productivity of society. Liver transplantation is the definitive treatment for patients with end-stage liver disease, improving their survival and quality of life. However, chronic rejection of the graft, impaired liver regeneration in these patients, and the imbalance between the demand for and the availability of organs compromise the effectiveness of this therapeutic strategy.

As a result of these challenges, there is an urgent need to find new diagnostic and therapeutic alternatives for these patients to block disease progression and prevent organ failure and associated comorbidities, as well as the subsequent socioeconomic costs.

In this context, a significant number of studies have demonstrated that protein kinase b (Akt) is a crucial factor in the regulation of liver function. For instance, liver cirrhosis has been associated with impaired Akt activity in experimental rat models, while the restoration of its function using gene therapy has improved liver hemodynamics [2]. Several mechanisms contribute to Akt dysregulation in liver dysfunction. Among these mechanisms, the association between G protein-coupled receptor kinase 2 (GRK2), an inhibitor of G protein-coupled receptor signaling, and Akt-mediated endothelial nitric oxide synthase (eNOS) activation has been reported in experimental models of liver fibrosis [3].

The beneficial effect of Akt activation on liver function was corroborated by a study conducted with an experimental model of orthotopic liver transplantation in pigs. In this case, ischemia-reperfusion injury during transplantation was the leading cause of oxidative stress and cellular death, and this outcome is clinically associated with loss of graft functionality and increased risk of graft rejection. Constitutive activation of the Akt pathway through the forced expression of a myr-Akt variant in transduced liver grafts restored liver function and counteracted apoptotic processes, increasing the viability of the graft [4]. This result supports previous findings showing the importance of Akt signaling in the maintenance of fundamental physiological processes of the liver.

Recent studies also suggested that Akt has an essential function in the liver regeneration process. Several studies have demonstrated that during liver regeneration, Akt is phosphorylated (active) and prevents apoptosis of hepatocytes by inducing cell survival signaling [5, 6]. Deficiency of Akt or its activating molecules, phosphoinositide-dependent protein kinase 1 (PDK1) and phosphoinositide 3-kinase (PI3-K), results in decreased liver regeneration and increased mortality after partial hepatectomy [7]. This outcome is the result of Akt and some of its targets or activators acting as positive regulators of cell survival, as in the case of insulin receptor (IR), insulin receptor substrate (IRS), PI3-K and phosphatidylinositol 3,4,5-trisphosphate 3-phosphatase (PTEN).

Additionally, Akt is a crucial mediator of cellular growth through direct regulation of mammalian target of rapamycin (mTOR). Ultimately, a study carried out using an experimental model of partial hepatectomy in forkhead box protein O1 (FoxO1) deficient mice demonstrated that specific inhibition of FoxO1 mediated by Akt in hepatocytes is essential for liver regeneration [8].

Notwithstanding all the evidence supporting the fact that Akt activity has an impact on the physiology and pathophysiology of the liver; most studies have been conducted in experimental rodent models. Thus, the activation status of Akt and its downstream targets in the liver of patients with chronic hepatopathy remain unknown. This lack of information limits the transfer of acquired knowledge to preclinical studies for development of new therapies or biomarkers. For this reason, the evaluation of the activation status of Akt and its molecular targets in patients with chronic liver disease is clinically relevant.

This study aimed to assess the activation status of proteins involved in the Akt/mTOR pathway in the livers of patients with cirrhosis. Additionally, we wanted to evaluate the association between Akt proteins and liver injury and loss of functionality, as well as determine their diagnostic and prognostic value.

## Materials and methods

### Study design and subjects

In this retrospective study, we included specimens from patients with or without cirrhosis (biopsy-confirmed in all cases) who underwent therapeutic partial hepatectomy. A total of 36 liver tissue samples were analyzed, of which 27 were cirrhotic tissue and 9 were healthy tissue (noncirrhotic group). The cirrhotic liver samples were obtained by liver resection from patients with cirrhosis associated with hepatitis C virus infection (HCV). The samples used for the “noncirrhotic group” were healthy liver tissue obtained from the resections of colorectal metastasis before vascular clamping. Samples with the histological presence of tumor tissue were excluded from further analysis. We have provided the demographic characteristics of the patients (age and sex) in Table 1.

**Table 1:** Demographic characteristics of patients included in this study.

Group	Noncirrhotic (n=9)	Cirrhotic (n=27)	Total	p-Value
Age, years Median (IQR)	51.0 (36.0–66.0)	66.5 (57.0–69.0)	65.5 (51.5–68.5)	NS
Sex % male	44.4 %	84.6 %	74.3 %	0.03

IQR, interquartile range.

### Analytical parameters of liver injury and function

In the cirrhotic patient group, several biomarkers of liver injury and functionality were evaluated to study their possible correlation with target proteins involved in the Akt pathway. For this purpose, biochemical markers were determined following the established clinical protocol: aspartate aminotransferase (ASAT), alanine aminotransferase (ALAT), gamma-glutamyl transferase (GGT), and albumin and bilirubin. The level of tumor marker alpha-fetoprotein (AFP) was also measured, along with other parameters included in a hepatic panel: glucose, cholesterol, and triglycerides. All parameters were determined in serum before liver resection surgery. All assays were carried out at the CORE laboratory of the Hospital Clínic of Barcelona using the autoanalyzers ADVIA 2400 Chemistry System and ADVIA Centaur XP (Siemens Healthineers, NY, USA). The FIB-4 score, which is a tool used to estimate the amount of liver fibrosis in patients with chronic hepatitis or cirrhosis was calculated based on four variables: patient age, serum levels of ASAT and ALAT, and platelet count in blood [9].

### Analysis of proteins involved in the Akt/mTOR pathway

We determined the levels of 10 phosphoproteins involved in the Akt/mTOR signaling pathway in a total of 36 liver tissue samples. The following analytes were quantified using a multiplex immunoassay based on Luminex^®^ xMAP^®^ technology, an Akt/mTOR phosphoprotein magnetic bead panel 11-plex 96-well plate assay (Merck Millipore, Darmstadt, Germany): Glycogen synthase kinase 3 beta (GSK3β), insulin like growth factor 1 receptor (IGF1R), insulin receptor substrate 1 (IRS1), mTOR, ribosomal protein S6 kinase beta-1 (p70S6K), IR, PTEN, glycogen synthase kinase 3 alpha (GSK3α), tuberous sclerosis complex 2 (TSC2), and ribosomal protein S6 (RPS6). This assay uses paramagnetic beads (6.5 μm diameter) to fix analytes and reaction substrates, allowing the quantification of multiple analytes in a single test. Assays were performed according to the manufacturer’s instructions; standards and samples were analyzed in duplicate, the incubation step was performed overnight with shaking at 4 °C (18 h, 750 rpm), and a hand-held magnetic block was used for the washing steps. Data were acquired with a Luminex MagPix 200 system (Luminex, Molecular Diagnostics, Toronto, Canada) and analyzed with XPonent software (Luminex, Molecular Diagnostics, Toronto, Canada).

For the liver tissue analysis, 100 mg of liver tissue was homogenized in lysis buffer (Tris-HCl 20 mM [pH 7.4] containing 1 % Triton X-100, 0.1 % sodium dodecyl sulfate, 50 mM NaCl, 2.5 mM ethylene diamine tetraacetic acid, 1 mM Na_4_P_2_O_7_·10H_2_O, 20 mM NaF, 1 mM Na_3_VO_4_, 2 mM Pefabloc and Complete; from Roche). Subsequently, the total protein concentration was quantified and normalized in all samples using a Pierce BCA protein assay kit (Thermo Fisher Scientific, MA, USA). The results are expressed as the median fluorescence intensity (MFI).

### Western blot analysis: expression levels of Akt and FoxO1 proteins

The tissue abundance of Akt and FoxO1 proteins, as well as their degree of phosphorylation, was determined by Western blot analysis. Tissue lysates were prepared in lysis buffer. Next, proteins were separated on a polyacrylamide gel with 10 % SDS (Mini Protean III; Bio-Rad, Richmond, CA) and transferred to 0.45-µm nitrocellulose membranes for 2 h at 48 °C. After blocking, the membranes were incubated at 48 °C overnight with the following antibodies: rabbit anti-FoxO1 (1:1,000; Cell Signaling), rabbit anti-Akt (1:5,000; Cell Signaling), rabbit anti-phospho-Akt (Ser473) (1:5,000; Cell Signaling) and anti-tubulin antibody (1:5,000 Cell Signaling). Next, the membranes were incubated with peroxidase-conjugated secondary antibodies at a 1:5,000 dilution (GE Healthcare) for 1 h at room temperature. The respective bands were visualized using Luminata Forte Western HRP Substrate (Millipore) and Image-Quant LAS 4000 (GE Healthcare). A densitometry analysis of the gels was performed using ImageJ software (version 1.37). The results are expressed in densitometric relative units (DRU) determined from the densitometric ratios of p-Akt/Akt and FoxO1/tubulin. To establish the quantitative linearity of the Western blot experiments, we used recombinant β-tubulin and recombinant total Akt 1 proteins at different concentrations (Recombinant human β-Tubulin protein-ab70187 and Recombinant human AKT1 protein-ab79792; Abcam).

### Statistical analysis

Continuous variables are expressed as the median ± interquartile range (IQR). Differences in quantitative variables among groups were evaluated using the Mann-Whitney *U* test. The degree of correlation between variables was determined using Pearson or Spearman coefficient, depending on the variable distribution. For linear correlation and linear regression analysis, the FoxO1 variable was logarithmically transformed (LnFoxO1) to correct the skewness of the distribution. The parameters that showed a significant or near significant correlation with analytical parameters of liver injury and function were selected for multiple regression analysis. The multivariate statistical analysis performed was linear regression. The validity of the resulting models was evaluated by plotting residuals or Pearson residuals against adjusted values and plotting the influence of each observation on the adjusted response according to Cook’s distance. All statistical analyses were performed using public libraries from the Comprehensive R Archive Network (CRAN; http://CRAN.R-project.org) in the open-source statistical computing environment R, version 3.1 (http://www.R-project.org/). GraphPad Prism version 8.0.2 software (GraphPad Software, CA, USA) was used for graphic representations. p-value ≤0.05 was considered significant. The statistical review of the study was performed by a biomedical statistician.

## Results

### Analytical parameters of liver injury and function

The demographic characteristics of the patients included in the study are shown in Table 1. It is important to note that although some research has suggested that FoxO1 may play a role in age-related diseases and lifespan, we did not find significant age differences (p=0.651) when comparing the two groups.

All cirrhotic patients exhibited elevated levels of aminotransferases (ALAT and ASAT) and GGT, which are indicative of liver injury, as shown in Table 2. These elevations in biochemical markers were observed solely in the serum samples from cirrhotic patients and not in those from the noncirrhotic patients.

**Table 2:** Analytical parameters determined in blood samples of cirrhotic patients.

Parameter	Reference range	Noncirrhotic (N=9)		Cirrhotic (n=27)		p-Value
		N	Median (IQR)	n	Median (IQR)	
ASAT	5–40 IU/L	8	22.5 (18.5–24.0)	25	71.0 (52.0–114.0)	<0.001
ALAT	5–40 IU/L	8	20.0 (15.5–23.0)	25	113.0 (72.0–165.0)	<0.001
GGT	5–40 IU/L	7	28.0 (14.0–45.0)	25	71.0 (38.0–140.0)	0.03
Albumin	34–48 g/L	7	43.0 (32.0–46.0)	24	43.0 (39.5–45.0)	NS
Bilirubin	<1.2 mg/dL	8	0.8 (0.60–1.0)	22	0.8 (0.6–1.0)	NS
Platelet count	130–400 × 10^9^/L	8	236.5 (206.0–286.5)	25	128 (99.0–162.0)	<0.001
Prothrombin time	80–100%	8	86.0 (76.0–97.5)	25	92.0 (86.0–100.0)	NS
Glucose	65–110 mg/dL	8	95.5 (88.0–120.0)	25	99.0 (87.0–119.0)	NS
Cholesterol	≤200 mg/dL	8	190.5 (176.0–207.5)	24	164.5 (149.5–180.5)	0.05
Triglycerides	<150 mg/dL	8	93.5 (66.0–102.5)	24	99.5 (86.5–135.0)	NS
Alpha-fetoprotein	<10 ng/mL	N.D.	N.D.	20	12.5 (7.5–49.0)	N.D.

Variables are expressed as the median and interquartile range. IU, international units; ALAT, alanin aminotransferase; ASAT, aspartate aminotransferase; GGT, gamma-glutamyl transferase; N, total number of patients in each group; n, number of patients for whom it was possible to determine the parameter; IQR, interquartile range; N.D., not determined.

### Comparative study: intrahepatic expression of Akt pathway proteins in cirrhotic patients vs. noncirrhotic patients

**Figure 1:**
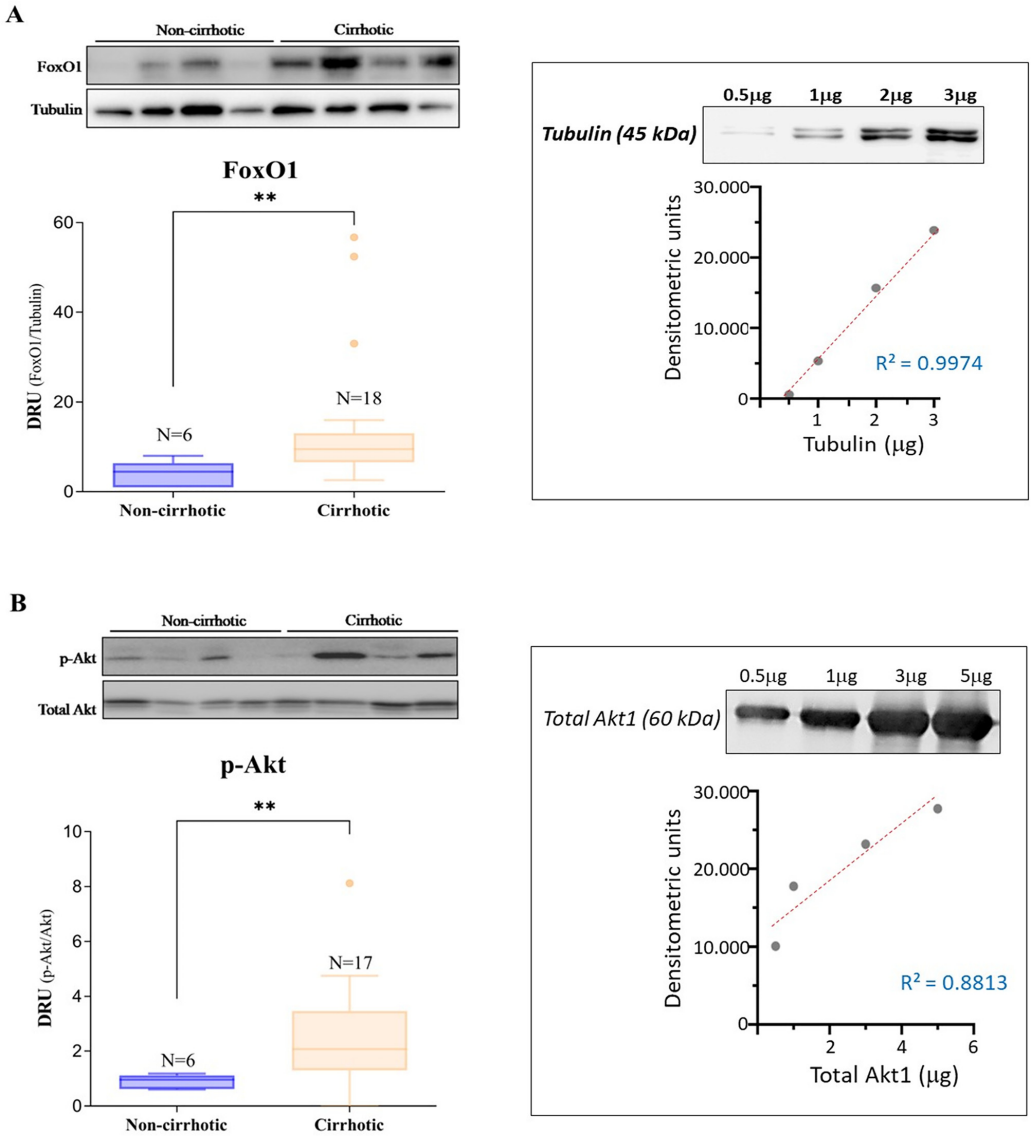
Expression of FoxO1 and p-Akt proteins in the livers of cirrhotic patients. Box plot comparing FoxO1 (A) and p-Akt (B) levels expressed as densitometric relative units (DRU). The DRU was calculated as the ratio between the specific FoxO1/tubulin and p-Akt/Akt bands quantified with ImageJ software. A representative image of Western blot analyses is shown at the top of each figure. Statistically significant differences (**p<0.01) were observed when comparing the median of both proteins between groups: FoxO1: median=4.4 (non-cirrhotic) vs. 9.5 (cirrhotic), p-Akt: median=1.0 (non-cirrhotic) vs. 2.1 (cirrhotic). The boxes on the right side of panels (A) and (B) show the quantitative capacity of the Western blots by demonstrating the linearity between the densitometric units measured and the increasing concentrations of recombinant proteins for tubulin and total Akt1, which were chosen as loading controls. The regression lines obtained from the recombinant proteins showed an acceptable R2.

Regarding protein expression levels of FoxO1 and Akt in liver tissues, the results showed increased levels of p-Akt (active form) in the cirrhotic patient group compared to the noncirrhotic group. These differences were statistically significant (p<0.01; see Figure 1A). In contrast, no statistically significant differences were found in Akt levels between the studied groups. The levels of FoxO1, which is negatively regulated by Akt, are presented in Figure 1B. The median FoxO1 level was significantly higher in the cirrhotic group than in the noncirrhotic group (p<0.01).

Among the target proteins analyzed by Luminex^®^—GSK3β, IGF1R, IRS1, mTOR, p70S6K, IR, PTEN, GSK3α, TSC2 and RPS6—statistically significant differences were observed for two proteins: p70S6K and PTEN.

**Figure 2:**
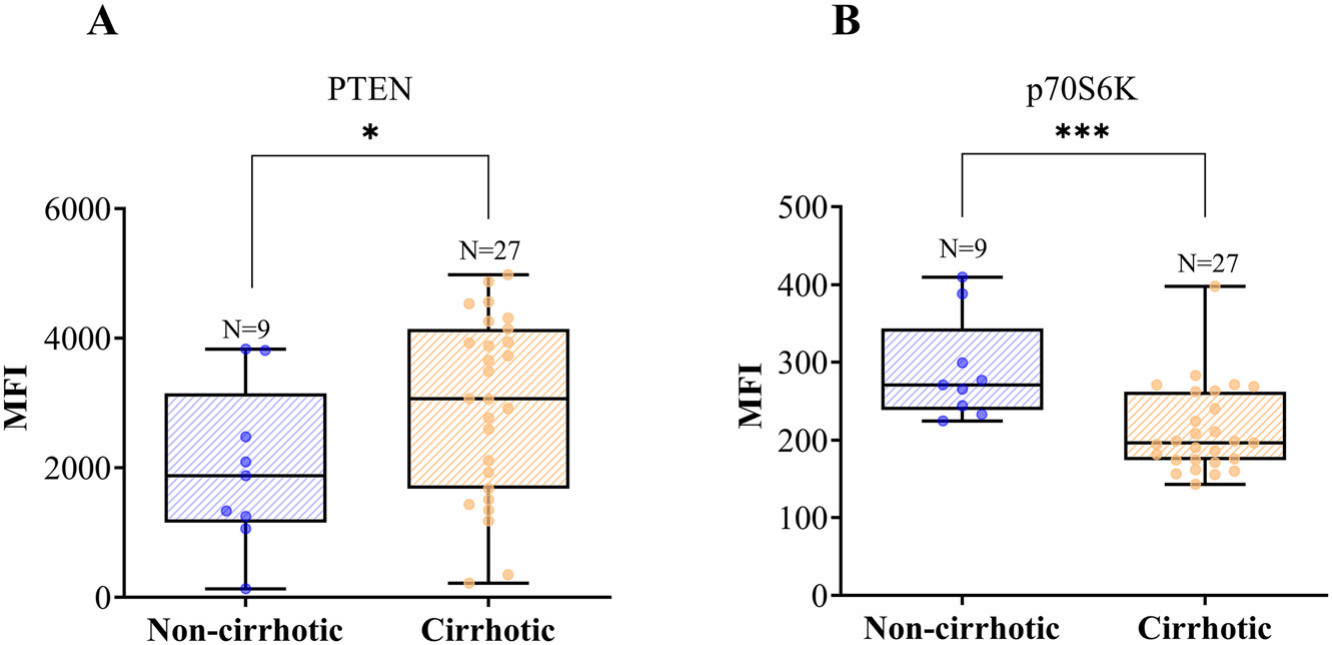
Box plot representative of PTEN (A) and p70S6K (B) levels in both groups. The levels of each protein in liver tissue are expressed in MFI (median fluorescence intensity). A: PTEN levels were significantly lower in non-cirrhotic patients (median=1877, n=9) compared to patients with cirrhosis (median=3,061, n=27) (*p<0.05). B: Non-cirrhotic patients had significantly higher levels of p70S6K (270.5; n=9) compared to cirrhotic patients (196.3; n=27) (***p<0.001).

According to the findings presented in Figure 2, the cirrhotic group demonstrated a significant increase in median PTEN levels compared to the noncirrhotic group (p<0.05). In contrast, the patients with cirrhosis displayed a notable decrease in p70S6K levels compared to those without cirrhosis (p<0.01).

No significant differences were found for the remaining variables when comparing the median between both groups (Table 3).

**Table 3:** Comparative study of 12 target proteins in the Akt pathway.

Parameter	Noncirrhotic (N=9)		Cirrhotic (n=27)		p-Value
	N	Median (IQR)	n	Median (IQR)	
^a^FoxO1	6	4.4 (1.0 – 6.4)	18	9.5 (6.6 – 13.0)	<0.01
p-Akt	6	1.0 (0.6 – 1.1)	17	2.1 (1.3 – 3.5)	<0.01
^b^GSK3α	9	123.0 (25.5 – 144.8)	27	158.5 (102.8 – 232.3)	NS
GSK3β	9	73.5 (66.8–155)	27	83.5 (64.0 – 112.5)	NS
IGF1R	9	2.5 (0 – 6.5)	27	5.0 (0 – 10.5)	NS
IR	9	8.5 (0 – 11.0)	27	9.0 (3.8 – 14.5)	NS
IRS1	9	57.5 (32.5 – 73.5)	27	66.8 (44.5 – 96.5)	NS
mTOR	9	43.5 (34.5 – 60.3)	27	52.0 (36.3 – 83.0)	NS
p70S6K	9	270.5 (243.8 – 299.0)	27	196.3 (174.9 – 262.0)	<0.01
PTEN	9	1876.5 (1,245.0 – 2,479.8)	27	3,060.8 (1,676.5 – 4,142.3)	0.04
RPS6	9	103.3 (64.0 – 127.5)	27	97.3 (44.5 – 268.5)	NS
TSC2	9	105.5 (49.5 – 193.5)	27	162.5 (105.3 – 260.0)	NS

Differences between groups were evaluated by Mann-Whitney test. p-value ≤0.05 was considered significant. ^a^FoxO1 and p-Akt values correspond to densitometric relative units (DRU), calculated as the densitometric ratio between FoxO1/tubulin and p-Akt/Akt. ^b^The remainder of the proteins are expressed in median fluorescence intensity (MFI) units. IQR, interquartile range; N, total number of patients in each group; n, number of patients for whom it was possible to determine the parameter.

### Correlation between Akt pathway proteins and analytical parameters of liver injury and function

**Figure 3:**
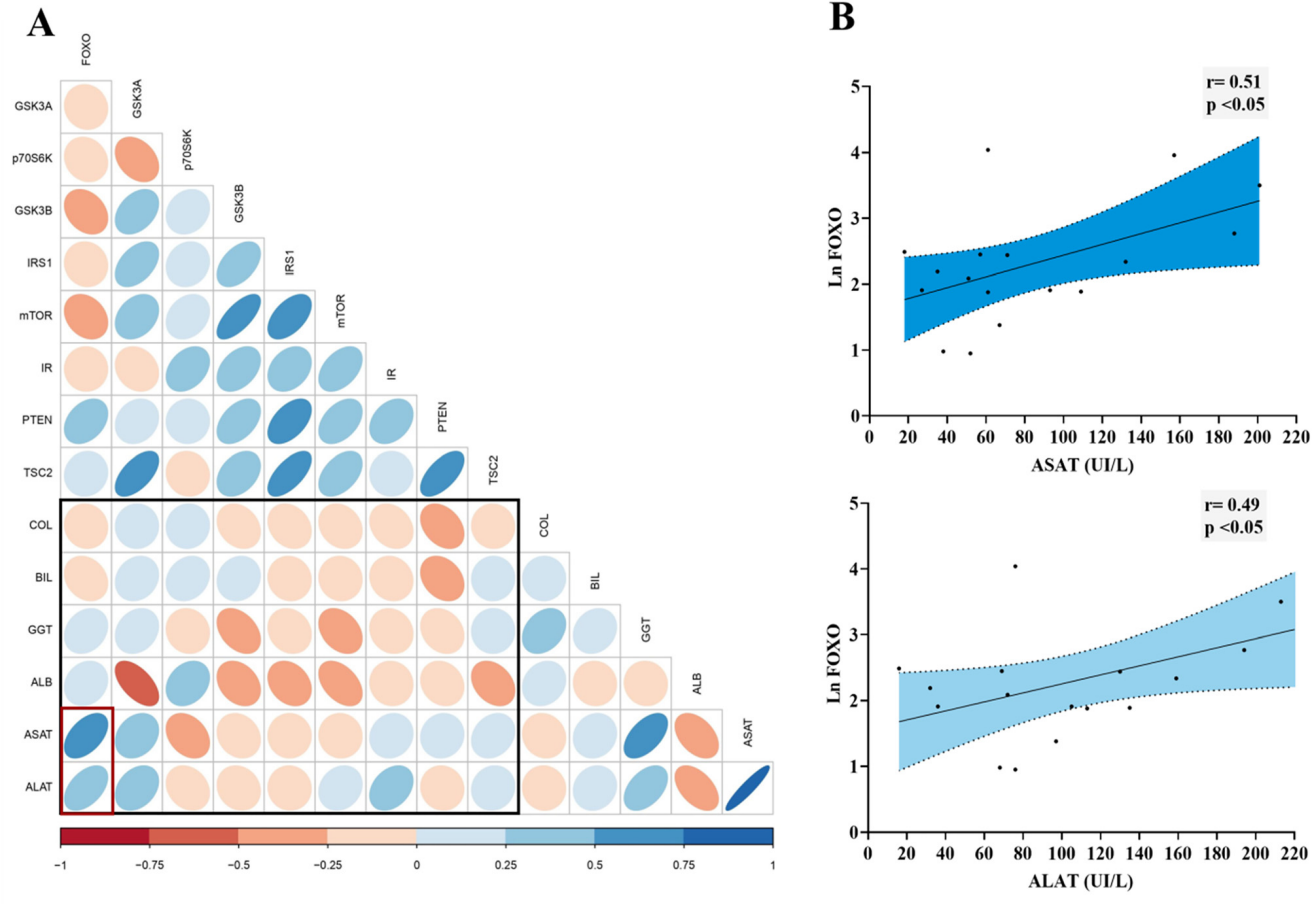
Correlation matrix between target proteins involved in the Akt pathway and analytical parameters of liver injury and function. (A) All possible correlations between the variables studied are shown. The black rectangle highlights the correlations of interest: the most relevant correlations between FoxO1 and transaminases are inside the red box. The color scale indicates whether it is a positive (blue) or negative (red) correlation. Color intensity and the size of the ellipses are proportional to the correlation coefficients (Pearson). (B) The scatter dot plot indicates that there is a positive correlation between the levels of FoxO1 protein in liver tissue and the concentration of transaminases (ASAT and ALAT) in serum. These correlations were statistically significant, with Pearson’s correlation coefficients: r=0.51 for ASAT and r=0.49 for ALAT. The corresponding regression line is also represented; the shaded area indicates the 95 % confidence intervals.

The association between all the studied proteins in the Akt pathway and the analytical parameters of functionality and liver injury was studied. Figure 3 shows the correlation matrix resulting from this analysis. The degrees of linear correlation, as well as significance, were calculated with Pearson correlation coefficients. Variables that did not fit a normal distribution were excluded from this matrix, and their relationships were evaluated individually by Spearman correlation coefficient. The expression of GSK3α is negatively correlated with the serum albumin level (r=−0.59, p=0.002) and positively correlated with ASAT (r=0.42; p=0.039). FoxO1 presented the best correlation with liver injury tests. The FoxO1 protein expression levels and serum concentrations of both transaminases showed a positive (moderate–high) and statistically significant correlation: ASAT (r=0.51, p=0.036) and ALAT (r=0.49, p=0.049). The linear correlation and the corresponding fitted regression line of FoxO1 and the transaminases are detailed in Figure 3B. Additionally, the expression of FoxO1 was found to have a moderate positive correlation with the FIB-4 score (r=0.46, p=0.036).

### Regression analysis explains the influence of FoxO1 on biochemical markers of liver injury

Since there was a statistically significant correlation between the concentration of transaminases in serum and the FoxO1 and GSK3α proteins, both Akt targets were included in the multivariate analysis, which showed that both parameters were positive independent predictors of ASAT concentration, explaining 37 % of the variance (R^2^-adj=0.37; F=5.75; p=0.015). In the case of the multivariate linear regression analysis of the dependent variable ALAT, only FoxO1 was identified as a positive independent predictor, explaining 18 % of the variance (R^2^-adj=0.18; F=4.61; p=0.049) (Supplementary Table 1).

## Discussion

The PI3K/AKT signaling pathway is crucial for energy homeostasis, cell growth, and survival. To date, studies in rodents have conclusively demonstrated that Akt plays a key role in liver regeneration as well as normal liver function [10]. The actions of the Akt pathway in the cell cycle are closely linked to the inhibition of FoxO1, a member of the Foxo (forkheadbox) transcription factors. These factors are necessary for multiple functions in the liver: they regulate the stress response, adaptation to fasting, and cell proliferation [11]. Akt inactivates FoxO1 by phosphorylation, which leads to its nuclear exclusion and thus inhibits its proapoptotic signaling [12]. For this reason, the Akt-FoxO1 interaction has been identified as the most important mechanism in liver regeneration. In summary, the maintenance of Akt activity and consequent inactivation of FoxO1 are associated with better functionality and regenerative capacity of the liver. Therefore, Akt and FoxO1 have been proposed as promising therapeutic targets for cirrhosis and other chronic liver diseases. However, there is currently insufficient evidence on the degree of activation of these molecules in cirrhotic patients, which hinders their possible translation to the clinic.

Our study is the first to comprehensively assess the activation status of Akt signaling pathway molecules in the livers of cirrhotic patients. Previously, we demonstrated in experimental models in cirrhotic rats that the enzymatic activity of Akt is decreased in the dysfunctional liver [2]. However, in the present study carried out in cirrhotic patients after partial hepatectomy, we showed that Akt and its target FoxO1 are overexpressed in liver cirrhosis. These conflicting results should be interpreted cautiously since these discrepancies may be attributed to other factors, such as comorbidities present in cirrhotic patients or the etiology of the disease. The latter is not a relevant factor in the animal model, in which cirrhosis is induced by carbon tetrachloride inhalation. In our cohort of patients with cirrhosis, the baseline etiology in all cases was HCV infection. The relationship between the Akt pathway and viral replication processes in chronic HCV infection is still unknown. *In vitro* studies suggest that the NS5A viral protein can activate the PI3K-Akt pathway, thus inhibiting cellular apoptosis [13]. Some authors have described this relationship as an evolutionary mechanism of most DNA mammalian viruses that promotes cell survival and ensures their replication [14].

The results of this study highlight the importance of validating the preclinical results obtained for the molecules involved in the Akt pathway in humans. The most relevant finding of the present study is that FoxO1 is overexpressed in the livers of cirrhotic patients. This finding is in line with previous research showing FoxO1 overexpression in liver tissue from patients with another chronic liver disease of different etiology, nonalcoholic steatohepatitis [15].

FoxO1 overexpression is clinically relevant considering that most patients diagnosed with HCC present with underlying cirrhosis [16]. Currently, surgical resection of these tumors or metastatic lesions in cirrhotic patients remains a significant clinical challenge due to the associated morbidity and mortality [17]. In cirrhotic patients, the regeneration capacity of hepatocytes after surgery is reduced, limiting the applicability of therapeutic resection in situations such as HCC [18]. This observation is consistent with the hypothesis that the higher the expression of FoxO1 is, the lower the liver’s regenerative capacity, as previously demonstrated in a murine model of liver regeneration caused by partial hepatectomy [8]. Furthermore, it is consistent with the fact that in our study, FoxO1 levels were significantly correlated with the degree of liver damage. Our statistical model revealed that, of the molecules studied, FoxO1 is the only candidate shown to be an independent predictor of ALAT and ASAT levels in serum. Therefore, considering both these results and our previous preclinical studies, FoxO1 overexpression may be one of the underlying mechanisms that limit liver regeneration in cirrhotic tissue. However, the primary limitation of our study is its sample size; therefore, these preliminary results should be validated through larger, multicenter prospective studies. The confirmation of this hypothesis is of great importance since the intrahepatic levels of FoxO1 may be an indicator of individual liver regenerative capacity and, therefore, a marker with prognostic value for patients with cirrhosis and HCC. In this context, understanding the mechanisms of induction and regulation of liver regeneration is needed to open the possibility of identifying new therapeutic targets. It would facilitate the development of therapies based on the restoration of liver functionality, improving the prognosis of patients with advanced liver disease.

Our results support the need to continue this line of research to better elucidate the role of Akt-FoxO1 in cirrhosis. Carrying out prospective observational studies is an appropriate strategy to determine the status of this pathway in the cirrhotic population with different etiologies. As a future line of research, it would be valuable to explore less invasive methods for studying the expression of this pathway than liver biopsies. Specifically, liquid biopsy strategies, such as the isolation of circulating exosomes from various biofluids and the analysis of their contents (i.e., RNAs, proteins and lipids), have gained attention as a promising approach for characterizing the genomic and transcriptomic profiles of liver diseases. Future studies may provide evidence to support the view that selective modulation of Akt or its targets is a viable strategy with beneficial effects for the management of patients with liver disease.

## Supplementary Material

Supplementary Material
