# Supplementary material for: Liver FoxO1 overexpression is positively associated with the degree of liver injury in cirrhotic patients
Source: Adv Lab Med. 2023 Jul 12;4(3):218–26. doi: 10.1515/almed-2023-0014 (PMC10701502; doi:10.1515/almed-2023-0014)
Supplement: Supplementary file 1 — Supplementary Material [file j_almed-2023-0014_suppl_001.docx]

SUPPLEMENTARY MATERIAL

Table 1. Multiple regression analysis results.

| Dependent variable  ASAT | F(2.14)=5.75 R^2^=0.45 R^2^ -adj=0.37 p-Value=0.015 | | | | |
| --- | --- | --- | --- | --- | --- |
|  | Coeff. | Err. Std | β | p-Value | 95% Confidence interval |
| Constant | -52.98 | 41.72 |  |  | -142.46 ̶ 36.51 |
| FoxO1 | 34.07 | 12.41 | 0.54 | 0.016 | 7.47 ̶ 60.69 |
| GSK3A | 0.38 | 0.17 | 0.44 | 0.045 | 0.01 ̶ 0.75 |
| Dependent variable  ALAT | F(1.15)=4.61 R^2^=0.24 R^2^-adj=0.18 p-Value=0.049 | | | | |
|  | Coeff. | Err. Std | β | p-Value | 95% Confidence interval |
| Constant | 28.20 | 39.25 |  |  | -55.45 ̶ 111.86 |
| FoxO1 | 34.20 | 15.96 | 0.49 | 0.049 | 0.25 ̶ 68.30 |

ASAT: aspartate aminotransferase, ALAT: alanin aminotransferase.
